# Understanding of HIV cure research in a rural community with high prevalence: the case of uMkhanyakude district, KwaZulu-Natal, South Africa

**DOI:** 10.1186/s12981-025-00806-9

**Published:** 2025-10-17

**Authors:** Rujeko Samanthia Chimukuche, Miliswa Magongo, Qinisile Shandu, Ingrid V. Bassett, Thumbi Ndung’u, Janet Seeley

**Affiliations:** 1 Africa Health Research Institute, KwaZulu-Natal, Nelson R. Mandela School of Medicine, 3rd Floor, K-RITH Tower Building, 719 Umbilo Road, Durban, Private Bag X7, Congella 4013 South Africa; 2 Division of Infection & Immunity, University College London, London, UK; 3 Division of Infectious Diseases, Massachusetts General Hospital, Boston, MA USA; 4 HIV Pathogenesis Programme, The Doris Duke Medical Research Institute, University of KwaZulu-Natal, Durban, South Africa; 5 Massachusetts Institute of Technology, Ragon Institute of Mass General Brigham, Harvard University, Cambridge, MA USA; 6 School of Nursing and Public Health, University of KwaZulu-Natal, KwaZulu-Natal, Durban, South Africa; 7 Department of Global Health and Development, London School of Tropical Hygiene and Medicine, London, UK

**Keywords:** HIV cure, Research, Public engagement, Public understanding, South Africa

## Abstract

**Background:**

Curing HIV has become a scientific priority with the development of HIV cure-related research collaborations and increasing clinical efforts. However, for potential study communities the meaning of HIV cure-related research needs to be fully understood.

**Methods:**

We conducted qualitative research in rural KwaZulu-Natal, South Africa investigating the knowledge and understanding of HIV cure. We used deliberative approaches to facilitate in-depth discussions. Five deliberative group discussions were conducted in IsiZulu (the local language) with five participants in each group. Data were audio-recorded and translated verbatim and transcribed into English in anonymised format. Data were later analysed thematically with three main themes identified: knowledge of HIV cure, HIV cure terminology and HIV cure trials.

**Results:**

Our findings showed that participants had a limited understanding of HIV cure-related research, a lack of trust regarding HIV cure science and participating in future cure trials. There were no local language terms used to describe HIV cure terminology, although several suggestions were shared in the discussions.

**Conclusions:**

Understanding the level of knowledge of rural populations regarding HIV cure-related research is essential for tailoring research and intervention strategies that meet their specific needs and circumstances. This can increase participation in the research and inform future HIV cure strategies.

## Background

Scientific advances have continued looking for an HIV cure. These have brought a range of promising durable antiretroviral therapy-free control strategies for HIV, which hold the potential to contribute to the removal of the need for lifelong antiretroviral therapy (ART) among people living with HIV [1]. As the number of people who live with HIV is expected to increase because of the uncertainties in a sustained HIV response [2, 3], the need and drive for an HIV cure has intensified. An HIV cure would not only address the health burdens of taking lifelong ART but would limit new infections and could ultimately significantly reduce HIV as a global health problem [4].

There are two definitions of HIV cure, eradication which refers to complete clearance of the virus from the body. This has been accomplished in at least seven cases. The most famous case is that of Timothy Ray Brown (the Berlin patient) who underwent a bone marrow transplant from a CCR5delta32 donor [5]. He became HIV seronegative. Remission, also referred to as functional cure, is another definition of cure. This is also referred to as durable ART-free control-which has been seen in elite controllers and some post-ART treatment controllers [6]. HIV cure-related research can be described as involving the scientific processes and studies aimed at finding a cure.

Despite ongoing HIV cure-related research conducted in many different settings globally, including in Africa [7–9], research with people living in rural settings with high HIV prevalence has been limited. Social science research in the Global North on HIV cure-related research has highlighted preferences, motivations, and the perceived risks in HIV cure clinical trials; similar data are required from the communities most affected by HIV in sub-Saharan Africa [10–13].

To set the stage for HIV cure-related research, it is important to engage with community members to gain an understanding of their knowledge about HIV cure science and attitudes towards HIV cure-related research. In places where many people are living with HIV, there could be considerable benefit derived from cure interventions such as sustained HIV remission, suppressed HIV viraemia, reduced transmission, prevention of re-infection, and maintaining viral control in the absence of ART [4]; therefore, expectations of the research may be high. Equally, there may be considerable apprehension about participation in trials of experimental cure technologies. Moreover, HIV cure-related research involves more than participation in clinical trials, with most efforts focused on exploratory and discovery work to inform HIV cure strategies.

A significant gap in Africa is the public understanding of concepts or terminology used in the field of HIV cure-related research such as eradication (permanent cure) or sustained remission off ART [9]. A study conducted in Ghana showed that a lack of understanding regarding HIV cure concepts contributed to the unwillingness of people living with HIV to consider taking part in HIV cure-related research [14].

In this paper we describe the findings from foundational research to assess the public understanding of HIV cure-related research, among a rural population in uMkhanyakude District, KwaZulu-Natal, South Africa, where the burden of HIV is high [15]. Using deliberative methods, we explored the understanding and usage of HIV cure terminology to understand the level of knowledge in this rural setting and build on this in co-creating HIV cure communications tailored for this population.

## Methods

### Theoretical framework

**Fig. 1:**
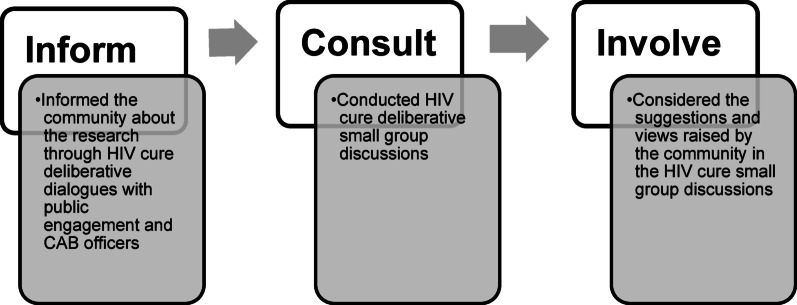
Adapted community-based participatory research (CBPR) framework in the communication of HIV cure-related research

This study used the community-based participatory research (CBPR) framework to formulate the deliberative small group discussion questions and guide our data analysis. CBPR is a collaborative approach to research that involves all partners in the research process equally, and prioritizes the involvement of the community in shaping and conducting research that directly impacts their lives [16]. Using the CBPR framework shifts from an approach where external researchers dictate the questions asked, the tools utilized, and the interventions needed to applied and participatory research (Fig. 1) [17]. Participatory research integrates the theoretical and methodological expertise of researchers with non-academics, encouraging a partnership [17].

In this study, our first step in reaching out to the community of uMkhanyakude was to engage with members of the community who work as public engagement officers and the Community Advisory Board (CAB) members at Africa Health Research Institute (AHRI) (Fig. 1). We conducted HIV cure deliberative dialogues sharing information on HIV cure science and building knowledge on the topic. Deliberative approaches incorporate informed discussions (where participants receive information on the topic to be discussed prior to the discussion), structured facilitation, and the encouragement of active participant engagement in questioning and discussing complex topics [18, 19]. This approach assisted us in informing the communities and involving them in this research process.

### Study setting

This study was conducted in uMkhanyakude District in northern KwaZulu-Natal Province, where HIV prevalence is 30% for individuals aged 15–49 years [20]. The district is largely made up of rural communities with traditional structures and leadership, which inform and shape the local value systems and norms [21, 22]. A significant percentage of the population in this district seeks care from traditional healers before turning to medical facilities [23, 24]. 55% of the population has primary school education or less, 19.9% of the population aged 20 years and above has no schooling, while 6% had attained higher education and 28% had completed secondary school. 35% live in the lowest financial stratum with 4% having no income at all [21].

### Study population

We conducted deliberative small group discussions from December 2024 to March 2025 with peer navigators (youth leaders working with the Africa Health Research Institute), aged 18–25 years [25], young adults aged 18–25, older age groups aged 45–60 years, traditional and church leaders and healthcare workers (including professional and enrolled public health nurses) (Table 1). Participants were purposively sampled and recruited through existing AHRI community engagement networks to ensure representation across different groups and communities. The HIV status of participants was not known and was not a selection criterion.

### Data collection

We used deliberative group discussions performed by a female trained facilitator as the primary method of data collection [26]. Deliberative approaches involve facilitated, in-depth discussions that allow participants to collectively learn and make sense of complex issues [19, 27]. Five deliberative discussion sessions took place in settings where the participants were comfortable. Each session began with 20–30 min of information sharing led by the facilitator [26], during which she explained what HIV cure-related research was and the different types of research that are being carried out. Participants were then asked a series of open-ended questions aimed at promoting discussion and exploring their understanding and attitudes towards HIV cure-related research.

The discussions were conducted in isiZulu, a local language, and were unstructured allowing flexibility in the way they evolved over the course of the session. Deliberative discussion summaries were written up immediately after each session. Data were audio-recorded, transcribed verbatim and translated into English and stored on a password-protected data server. These summaries provided an overview of the conduct of the group discussion and the main points raised in order to complement the transcription after processing. Each translated transcript was quality checked for accuracy.

### Data analysis

The data analysis was conducted using a thematic approach [28]. Initially, the research team familiarized themselves with the translated transcripts. The coding framework was developed by the first author from the data that the entire team had reviewed. The main codes were reviewed, discussed and agreed on by consensus for validity, and similar codes were merged to avoid duplication. Then the research team undertook analytical coding manually to identify recurring patterns and meanings within the data. The team then reviewed and refined these codes, grouping them into broad key themes. Three main themes emerged from this process: knowledge of HIV cure, HIV cure-related terminology, and attitudes towards HIV cure-related clinical trials. These themes were then charted using Excel to compare and synthesise. To document reflections and insights, the team drafted analytical memos reflecting on the emerging themes throughout the analysis. To ensure rigour, team members implemented a multi-stage quality checking process whereby members compared their coding and discussed their individual reflections from the analytical memos.

The occurrence of the main themes is summarised in the following table:

**Table 1:** Frequency of occurrence of main themes in discussions

Group category	Knowledge of HIV cure	HIV cure-related terminology	HIV cure-related trials
AHRI peer navigators	Frequently	Frequently	Not mentioned
Community young adults	Frequently	Not mentioned	Frequently
Traditional and religious leaders	Frequently	Frequently	Frequently
Older age group	Frequently	Frequently	Frequently
Health care Workers	Less frequently	Less frequently	Less frequently

## Results

A total of five deliberative small group discussions were conducted with five participants in each group (Table 1). The groups included AHRI peer navigators (18–25 years), young adults from the local community (18–25 years), traditional and religious leaders (aged between 50 and 72 years), an older age group of community members (40–60 years) and health care workers (Table 2).

**Table 2:** HIV cure small group discussion demographics

Group category	Sex	Age range	Description
AHRI peer navigators	3 females 2 males	18–25 years	Youth leaders working with the Africa Health Research Institute, selected by community leaders from 21 HIV prevention programme implementation areas in uMkhanyakude to provide support to young people to access the programme intervention
Community young adults	2 females 3 males	18–25 years	Young adults recruited from the Africa Health Research Institute health demographic surveillance site in uMkhanyakude district
Traditional and religious leaders	2 females 3 males	50–72 years	Traditional and religious leaders from the community of uMkhanyakude district
Older age group	3 females 2 males	40–60 years	Older community members of the community from uMkhanyakude district
Health care Workers	4 females 1 male	37–48 years	Three enrolled nurses, one registered nurse and an operational manager

### Knowledge of HIV cure

Participants highlighted their acquired knowledge of HIV cure derived from the deliberative process, interpreting HIV cure as eradication of the virus from the body:

*I believe we are talking about HIV*,* so the cure would target the HIV root causes rather than just addressing symptoms or related diseases. It would completely eliminate HIV at its source*,* eradicating the virus itself rather than focusing on any specific part of it.* Female, 24 years old (AHRI peer navigators).

Participants stated that a cure for HIV would not only transform the lives of those already living with the virus, but play an important role in global public health by preventing further transmission and reducing new cases:

*Finding an HIV cure is very important. It would reduce the number of new acquisitions and thereby protect those who are currently not living with HIV. Additionally*,* it would lower the chances of transmission from people living with HIV.* Female, 25 years old (AHRI peer navigators).

Some participants emphasised emotional and social barriers that result from ongoing HIV treatment, such as fear, stigma, or access to care. An HIV cure would represent more than just a medical breakthrough—it would offer relief from the burdens of daily treatment and healthcare visits as expressed by a young adult:

*I think it is important to have an HIV cure medication because it would help people who are afraid of going to clinics to get their medication. It would be much better if the virus could be entirely eradicated.* Male, 25 years old (Community young adults).

Some participants stated that a cure represents not only medical progress but also emotional relief and the chance to live without fear of transmission or stigma. These participants said that a sense of hope is especially meaningful for older adults who have lived through the evolution of HIV treatment. A religious leader stated:

*HIV will eventually become like a headache*,* something you can cure yourself of. Look at the London and Berlin patients. We learned about the bone marrow transplant. If the virus is suppressed*,* they need to eliminate it completely from the body so that even if someone has been living with HIV*,* they can no longer pass it on to the next person. HIV will be eradicated because scientists are continuing their research*,* and that gives us hope.* Female, 55 years (Traditional and religious leaders).

Nearly all the participants said that they had limited knowledge of current scientific efforts to find an HIV cure. This lack of knowledge led to questions about the effectiveness and implications of such research since the description given at the beginning of the sessions had introduced information that was completely new to the discussants. Participants had many questions. One 25 year old man raised concerns about reinfection and the long-term reliability of a cure: *Once a person is cured*,* what happens if they are exposed to HIV again? Will people ever be able to contract HIV again? Once you’re cured*,* will the HIV ever come back?* (AHRI peer navigators).

Lack of trust in HIV cure-related information currently accessible on social media or cure remedies offered by religious and traditional healers was expressed by the older groups. Scepticism in this information was seemingly influenced by being a part of a generation that experienced the evolution of HIV as a condition and the HIV prevention methods and treatment. The participants shared stories of witnessing individuals die after trusting unverified HIV cures. These people therefore have a certain level of mistrust in HIV cure information which may be a barrier to future HIV cure engagements.

*We don’t have any knowledge of an HIV cure. The people who talk on the radio*,* it’s not because they know. They just know that we are sick*,* so when a person is sick*,* some people play with them…I don’t trust the people they invite on the radio because they say they have a cure; they give it to you*,* then you give them the money. People then stop taking their pills and keep getting worse because they want money and not to help people.* Male, 63 years old (Older age group).

Even among the younger participants, while some were hopeful due to recent health advancements, they knew little about cure science:

*We are hearing for the first time that HIV can be cured. We grew up knowing that HIV couldn’t be cured. But when we look at the progress the Department of Health has brought to the community… I don’t think it will be difficult.* Male, 25 years old (AHRI peer navigators).

Traditional healers acknowledged the value of HIV cure-related research because of the difference between HIV and other ailments:

*No*,* I don’t think HIV can be cured as easily as a headache. As a Zulu person*,* you can cure a headache using indigenous herbs just by inhaling them*,* and you’ll feel fine. But HIV is different; it’s not something that can just disappear like that. This person needs the research you are conducting here.* Male, 54 years old (Traditional healer).

The traditional healers went on to highlight that even in their practice they had not seen a cure;

*No*,* I don’t know anyone who can cure HIV because even the ones who claimed they can cure it. They have not shown us proof that they can completely eradicate it*,* except for the treatment you get from the clinic.* Male, 54 years old (Traditional healer).

Participants in this study expressed and accepted that traditional medicine does not cure HIV and that HIV cure-related research was valuable.

### HIV cure terminology

When participants were initially asked how they describe HIV cure in their language (isiZulu) the general response was that there is no such a term in isiZulu. However, drawing from the knowledge they had gained during the deliberative session participants suggested terms in the local language that could be used to describe HIV cure. Some stressed the eradication of the HIV virus, *I think the words that have deep meaning that we can use include umkhucululazonke (HIV eradicator).* Female, 25 years (AHRI peer navigators). Whilst others stated that HIV cure can be termed *Zifozonke –an HIV cure will cure the virus.* Female, 47 years old (Older age group).

The IsiZulu terms that were suggested described the understanding that participants had of HIV cure. Some participants further suggested terms reflecting that an HIV cure would lead to fewer deaths as stated by an older participant: *We would call it “umgcwabo uhlehlile* (which means no one has to die prematurely because of HIV/AIDS). Male 43 years old (Older age group).

On the other hand, health care workers suggested terms that described the importance of the HIV epidemic coming to an end:

*Oh mix the two words “umqa… umqamulaj’uqu wobhubhane* (meaning the end of the epidemic). *We’ve come a very long way looking for a cure. So many people died before we even got the treatment that suppresses it. And now*,* finally*,* we have something that could actually end it.* Male 37 years old (Health care worker).

### HIV cure-related trials

Discussions took place around motivations and barriers towards participation in future HIV cure-related clinical trials. Participants expressed mixed views regarding participation, especially in the communities where they reside: *It would be a great thing to participate in HIV cure trials. However*,* I can also foresee a problem where people might behave recklessly in our communities*,* knowing that there is now a cure.* Female, 25 years old (AHRI peer navigators). The participant expressed risky socio-behavioural consequences that could arise if the HIV cure messaging is not communicated carefully and clearly to the public.

Stigma was highlighted as one of the barriers to HIV cure-related clinical trial participation in this setting with young participants highlighting that it will be better if these trials were done far away:

*I would not take part in an HIV cure clinical trial because*,* in doing so*,* I would have to disclose my status to help others. Unfortunately*,* many people still lack proper education about HIV. If I were to have a child*,* they might face discrimination simply because their parents were living with HIV. They would grow up carrying the burden of a stigma.* Male, 23 years old.

The traditional and religious leaders highlighted fears around participation in HIV cure-related research, and HIV-related research in general. This may hinder the willingness to take part since there will be doubts about the effectiveness of the trial processes and the risks participants will be exposed to.

*Even with this*,* we will be happy*,* but there is fear and trauma. You would start thinking*,* “What if*,* after getting the vaccine*,* I don’t wake up in the morning? What if I don’t wake up after taking the pill? Were people just using me to do their tests?” There will be mixed emotions*,* both fear and happiness.* Female, 71 years old (Traditional and religious leaders).

Engaging communities using the CPBR framework in the HIV cure small group discussions showed the importance of knowledge sharing (Fig. 1). Communities can co-develop HIV cure terminology that is contextualised as in the suggestions above. Further, the deliberative dialogues provided a first step in involving all partners equally in the research process by engaging them in their views and expectations in research that directly impacts their lives.

### Key implications

From the results of this study, we have identified some key implications of this research in uMkhanyakude, South Africa. We summarise these in Table 3, below.

**Table 3:** Key implications

Theme	Implications
Knowledge of HIV cure	Increased understanding of HIV cure related research in populations that have low education levels.
HIV cure terminology	Tailored language on HIV cure terms increases understanding on HIV cure.
HIV cure trials	Increased participation in clinical trials Clear risks and benefits of participating are communicated

## Discussion

Study findings show that while participants demonstrated knowledge of HIV prevention and treatment there is still a large gap in knowledge about HIV cure-related science. This lack of knowledge increases the lack of trust regarding the HIV cure-related science and participating in the cure trials. Lastly, HIV cure terminology can be contextualised in terms that communities can understand and help to co-create that terminology.

In this study, the use of deliberative methods was effective, as it allowed participants time to reflect on the information presented and engage meaningfully with the topic. Participants reflected a positive attitude and excitement at the prospect of an HIV cure. The different groups of participants shared their hopes that a cure would reduce new infections, alleviate the burden of taking ART, and help eliminate HIV-related stigma. These are similar sentiments shared in other studies such as the FRESH trial where participants also see an HIV cure as a means of addressing current HIV socio-related issues [29].

Similar to other studies conducted in lower and middle income countries, our results show that there is still limited knowledge and understanding about HIV cure in the general population in South Africa [9, 30]. While participants across all groups showed a limited understanding of HIV cure, they all expressed the importance of finding a cure for HIV. Most of them understood cure as a complete eradication of the virus from the body, which aligns with perceptions from other studies [7, 29, 31]. This means that in future engagements, researchers will need to find strategies that clearly articulate the different types of cures and also come up with strategies to manage community expectations to avoid disappointment or misunderstanding.

Notably, participants in this study emphasized the need for a cure that completely eradicates HIV. Although this outcome is desirable and is the ultimate goal of HIV cure research, there is a need to balance this goal with the more achievable one of durable ART-free transmission, where the virus is suppressed below the presumed HIV transmission threshold of 200 copies/ml as envisioned in recently published target product profiles [32, 33]. According to the World Health Organisation, three key categories for HIV viral load measurements are recognised: unsuppressed (> 1000 copies/mL), suppressed (detected but ≤ 1000 copies/mL) and undetectable (viral load not detected by test used) [34]. Further engagement of communities on this nuanced pathway towards a cure for HIV is needed.

Our results showed that there is no isiZulu word for “HIV cure,” suggesting that the concept is still new and unfamiliar in the local language. In this study, the deliberative process was helpful in showing how participants, when supported by well-facilitated discussions, can suggest terms for these unfamiliar concepts [19, 26]. Although participants did not offer elaborate explanations in isiZulu of HIV cure science, this first approach of identifying local terms offers a useful step in developing a strategy for researchers seeking to develop culturally appropriate and locally relevant language for communicating complex scientific concepts like HIV cure in rural communities [26, 27].

Our findings show the potential for collaboration with traditional healers in HIV cure-related research. Traditional healers are deeply embedded in the South African communities and are often the first place that individuals go to seek healthcare. The traditional healers we engaged with recognised that HIV is fundamentally different from illnesses they usually treat with herbs, acknowledging the need for biomedical intervention. However, a lack of trust in past unproven ‘cure remedies’ by traditional healers could fuel mistrust in HIV cure-related research. This has been shown in an ethnographic study that highlighted the influence of traditional healing on HIV and AIDS management in South Africa [35]. A shared understanding creates common ground between traditional and scientific approaches, as observed in a qualitative study in Mozambique, where engagement with HIV services improved by incorporating traditional healers in this endemic region [36]. This positions traditional healers as valuable partners in community education and HIV cure engagement efforts [37] .

In terms of clinical trial participation, our results showed mixed views about taking part in the future with some participants saying that they were motivated by a sense of being part of a process that could make a difference for future generations and their communities. This finding resonates with other research which highlights altruism as a key motivating factor for participation in clinical research [38]. However, other participants identified several potential barriers to trial participation from a community perspective. These included HIV-related stigma, negative attitudes from healthcare workers, and long waiting times at clinics, relating to other HIV-related studies describing the same factors as key obstacles to the uptake of HIV testing and care services [39].

### Limitations

Our research was conducted in a single rural district, therefore the generalizability of the findings may be limited and applicable only to our setting. This may not resonate with other settings that have different cultures, languages, and socio-economic contexts. Another limitation was the engagement with AHRI peer navigators—a group with experience in HIV research and more informed views and perspectives. While we also included input from local community youths, the generalizability of these methods may still be limited. That said, while the use of quantitative methods might allow for a broader comparison across populations, the use of qualitative methods allowed us to gather detailed information on the respondents’ questions about HIV cure and associated research. Therefore a strength of our qualitative approach lies in providing in-depth insights into the experiences and perspectives that would be difficult to capture using quantitative methods.

## Conclusion

Understanding the knowledge of rural populations regarding HIV vaccines and cure research is essential for tailoring research and intervention strategies that meet their specific needs and circumstances. By incorporating insights from social science research, researchers and healthcare providers can find out what HIV cure really means for Africans, what kinds of cure trial designs may be acceptable on the continent, and develop culturally sensitive approaches that promote acceptance and engagement with HIV prevention and treatment efforts in rural communities.

## Data Availability

The datasets used and/or analysed during the current study are available from the corresponding author on reasonable request.

## Abbreviations

AHRI: Africa Health Research Institute
ART: Antiretroviral Treatment
CAB: Community Advisory Board
CBPR: Community-based participatory research
LMIC: Low middle to income countries

## Data availability

The datasets used and/or analysed during the current study are available from the corresponding author on reasonable request.
